# Relationship Between Oral Motor and Cognitive Function in Community-Dwelling Korean Older Adults: A Cross-Sectional, Observational Study

**DOI:** 10.3390/healthcare12202097

**Published:** 2024-10-21

**Authors:** Nam-Hae Jung

**Affiliations:** Department of Occupational Therapy, Dongseo University, 47 Juryero, Sasan-gu, Busan 47011, Republic of Korea; whitenam-hae@nate.com

**Keywords:** cognition, dysphagia, older adults, oral motor function

## Abstract

**Background**: The present study analyzed the relationship between oral motor and cognitive functions in community-dwelling older Korean adults. **Methods:** Study participants included 113 community-dwelling older adults with a mean age of 73.84 years. Subjects’ cognition was assessed using the Korean version of the Montreal Cognitive Assessment, and tongue, cheek, and lip pressures were assessed using the Iowa Oral Performance Instrument. Tongue and masseter thicknesses were measured using an ultrasound device (Sonon, Healcerion, Roseville, CA, USA). The occlusal force was measured using a specialized device (Innobyte, Kube Innovation, Montreal, QC, Canada), and the number of lost teeth was directly verified by a dental hygienist. **Results:** Results of analysis of cognitive function according to demographic characteristics of older community-dwelling adults revealed a significant difference in cognitive function according to education level and employment status. Cognitive function demonstrated a positive correlation with oral motor function, including pressure on the cheek, lips, and tongue, thickness of the masseter and tongue, occlusal force, number of lost teeth, and age. Hierarchical regression analysis revealed that demographic characteristics and cheek, lip, tongue, and masseter functions did not affect cognition, whereas occlusal force and number of lost teeth significantly affected cognition. In this study, oral motor function, excluding occlusal force and number of lost teeth, did not affect cognition. **Conclusions:** Future studies, however, are required to analyze the relationship between oral motor function and cognition in older adults with a wider range of such functions.

## 1. Introduction

Dementia is a chronic or progressive syndrome that affects memory, thinking, behavior, and the ability to perform daily activities due to cognitive decline caused by neurodegenerative disease(s) [1]. The World Health Organization (WHO) projected that the prevalence of dementia will increase by 131 million by 2050 [2]. According to the WHO, dementia is one of the major causes of disability and dependency among the elderly and the seventh leading cause of death among all diseases. There is currently no cure for dementia; as such, approaches to prevent its onset and slow its progression are important [3].

Several studies have investigated masticatory function as a factor associated with dementia. Studies investigating the effects of the series of processes of chewing and swallowing on attention, memory, and cognitive processing have demonstrated that masticatory function is associated with cognitive function [4,5,6]. Objective and subjective methods are available for evaluating masticatory function. Objective evaluation can be divided into a direct method of chewing solid foods and an indirect method of measuring factors such as jaw movement, masticatory muscle activity, and occlusal force. Subjective evaluation can be performed using questionnaires [7]. Eagshira et al. [8] reported that related previous studies mainly used subjective evaluation methods, such as self-administered questionnaires, to evaluate masticatory function and that each study was not comprehensive and only evaluated limited factors. Maekawa et al. [9] reported that the correlation between chewing function and cognitive status using chewing gum, an objective and direct evaluation method, was ambiguous, whereas occlusal force exhibited a significant correlation with cognition. They reported that the reason for this was the complexity of the objective and direct measurements. Although occlusal force measurement, an objective and indirect evaluation method, is an indirect method that can be measured through a simple biting movement, evaluation using chewing gum, a pseudo-objective direct evaluation method, requires comprehensive and complex movements involving various organs such as the tongue, jaw, lips, and cheeks, making accurate measurement difficult. Although there are objective and direct methods for assessing chewing ability, objective indirect measurement methods may be preferred for a clear evaluation, due to practical limitations.

Previous studies have mostly analyzed chewing function using chewing gum and the number of teeth as masticatory variables in studies investigating the relationship with cognition [9,10,11]. Existing evidence regarding the relationship between oral motor function, including the lips, tongue, cheeks, and masticatory muscles, and cognitive status is weak, and more is needed [12,13].

Oral motor function plays an important role in functions such as swallowing, mastication, breathing, and speaking, and an important role in the oropharyngeal stage by forming a large pressure for lip closure, oral bolus formation, and propulsion into the pharynx [14,15,16]. Among these oral motor functions, it has been reported that the muscle strength of the cheeks and lips affects oral closure and manipulation and that the functional movement of the tongue facilitates mastication and swallowing [14]. Oral motor structures, such as the tongue, palate, cheeks, and lips, grind food particles between chewing surfaces to create a bolus, which alters the position between the teeth during the masticatory cycle [17,18]. Symptoms of dysphagia include impaired motor and sensory function of the tongue, difficulty in forming and moving food bolus, residual food remaining in the mouth after swallowing, delayed oral movement time due to weakness and incoordination of the tongue, lips, and facial muscles, and premature leakage and spillage of food before swallowing, aspiration, and invasion appear [19,20,21]. In addition, tongue muscle atrophy reduces tongue pressure and makes swallowing difficult [22]. As the importance of oral motor function in swallowing has been highlighted, studies have been reported that standardized the fiber composition of oral motor structures and tongue pressure, as well as studies investigating changes in tongue pressure during swallowing and improving tongue pressure and facial muscle function in patients with dysphagia [14,15,23]. Although many studies have addressed oral motor function and swallowing, there is a lack of research investigating the effects of oral motor function on cognitive function.

Among studies investigating the relationship between masticatory function and cognition, most assessment tools, such as the Mini-Mental State Examination (MMSE), Hasegawa Dementia Scale-Revised (HDS-R), and Frontal Assessment Battery (FAB), have been used to evaluate cognition [24]. The MMSE and HDS-R have similar score distributions and are administered to screen subjects with low cognitive levels. In contrast, the FAB is a representative screening tool for evaluating executive function [25]. In studies investigating the relationship between masticatory function and cognition, it is necessary to consider the ceiling effect of the MMSE and HDS-R, evaluate various cognitive levels, and use assessment tools that can evaluate not only executive function but also overall cognitive function. In addition, direct comparison through objective measurement of comprehensive variables is necessary to confirm the relationship between masticatory and cognitive function(s) [26].

As such, the purpose of the present study was to analyze the relationship between oral motor function―measured using objective and indirect evaluation methods for the cheek, tongue, lips, and masseter muscles―and cognition using the Korean version of the Montreal Cognitive Assessment (MoCA-K), which is used to evaluate overall cognitive function in older adults. 

## 2. Materials and Methods

### 2.1. Participants

The sample size for the study was calculated using the G*Power 3.1.9.4 program (Heinrich-Heine University, Düsseldorf, Germany) to determine the number of subjects required for hierarchical regression analysis. The minimum sample size was found to be 64, assuming an effect size of 0.35, a significance level of 0.05, a power of 0.80, and the analysis of 13 predictor variables. A total of 113 elderly individuals living independently in the community participated in this study. The inclusion criteria were age ≥65 years, a willingness to participate actively, adequate communication and cognitive abilities (capable of following study instructions), and being generally active, with the ability to sit unaided. Exclusion criteria included a history of speech or swallowing issues, parafunctional habits (such as excessive sucking or biting), trigeminal neuropathy, tooth pain, and severe malocclusion. The study protocol was approved by the Institutional Review Board of DongSeo University (Busan, Korea; 2024-018-HR-02) and all subjects provided informed written consent to participate. 

### 2.2. Measures

#### 2.2.1. Cognitive Function

Cognitive function was assessed by the occupational therapists using the MoCA-K. The MoCA-K consists of 7 items: visuospatial-executive, naming, attention, language, abstraction, delayed recall, and orientation. Scores range from 0 to 30, with higher scores indicating better cognitive ability, and scores over 23 indicating normal cognition. The test–retest reliability of the MoCA-K was 0.85, and concurrent validity with the MMSE-K was confirmed [27].

#### 2.2.2. Tongue Pressure and Thickness

Tongue pressure was measured by occupational therapists using the Iowa Oral Performance Instrument (IOPI Medical LLC, Redmond, WA, USA). Measurements were performed 3 times for each row, and the mean value was calculated and recorded. Tongue thickness was assessed using ultrasonography (Sonon 300 L, Healcerion, Roseville, CA, USA) with a 10 MHz linear and convex array transducer, measuring from the mylohyoid muscle to the tongue’s surface. Two measurements were taken when the tongue was at rest after swallowing saliva, and the mean value was calculated. 

#### 2.2.3. Cheek and Lip Pressure

Cheek and lip pressures were measured by occupational therapists using the IOPI according to methods described in a previous study. The bulb was placed between the lateral teeth and cheek, and the participants were instructed to gently close their mouths and squeeze the bulb for 2 s. To assess orbicularis oris muscle strength, the bulb was inserted between two disposable tongue depressors, aligned at the lip’s center. Participants gently closed their lips, slightly protruded them, and pressed the tongue depressor as hard as possible with their lips.

#### 2.2.4. Occlusal Force and Number of Lost Teeth

The occlusal force was measured using a specialized device (Innobyte, Kube Innovation, Montreal, QC, Canada) by occupational therapists. Participants were seated in a relaxed, upright position and instructed to bite down on a pressure sensor as hard as they could for 5 s. The average of three measurements was recorded for analysis, with the maximum occlusal force expressed in Newtons. A dental hygienist verified the number of lost teeth.

#### 2.2.5. Oral Motor Function (Masseter Thickness)

Masseter muscle thickness was measured using an ultrasonography device (Sonon, Healcerion) by the occupational therapists. The participants were instructed to remain in a seated position. Participants were seated, and both left and right masseter muscles were scanned during clenching. A linear transducer was set to 10 MHz and 66 dB for all participants. The transducer was positioned along the line between the external auditory meatus and acanthion and adjusted 2–3 cm downward to align with the outer canthus and masseter muscle, as previously reported. The thickest part of the masseter muscle was used to determine its thickness.

### 2.3. Statistical Analysis

All statistical analyses were conducted using SPSS version 24.0 (IBM Corp., Armonk, NY, USA). Descriptive statistics were used to present the participants’ demographic information as means and standard deviations (SDs), and frequency analysis was also performed. MoCA-K scores according to demographic characteristics were subjected to an independent *t*-test and one-way analysis of variance (ANOVA). Post-hoc analysis was conducted using the Scheffe test. Correlation analysis was performed to determine the relationship between age, cognition, and oral motor function. A hierarchical regression analysis was performed to determine the effects of general characteristics, oral motor function, number of teeth lost, and occlusal force on cognition. Differences with *p* < 0.05 were considered to be statistically significant.

## 3. Results

### 3.1. Cognitive and Oral Facial Function 

In total, 113 elderly individuals participated in this study. The mean age was 73.84 years, and there were more females (67.3%) than males. The general characteristics of the subjects, information regarding the pressure on the lips and cheeks, and the pressure and thickness of the masseter and tongue are summarized in Table 1.

### 3.2. Cognitive Function According to General Characteristics

MoCA-K scores were analyzed according to general characteristics. MoCA-K scores for older individuals exhibited significant differences according to education level and employment status (*p* < 0.05). The MoCA-K scores in the elementary school group were significantly lower than those of the high school and university groups. There were no significant differences in MoCA-K scores according to sex, smoking, or alcohol consumption (*p* > 0.05) (Table 2).

### 3.3. Relationship Between Cognitive Function and Oral Motor Function

The MoCA-K scores of older adults were analyzed for correlations with variables related to oral motor function and age. The MoCA-K score was negatively correlated with age and tooth loss and positively correlated with occlusal force, tongue pressure and thickness, masseter thickness, and cheek and lip pressures (*p* < 0.05). As age and tooth loss increased, MoCA-K scores decreased, and as occlusal force, tongue pressure, and masseter muscle thickness and cheek pressure, and lip pressure increased, MoCA-K scores increased (Table 3).

### 3.4. Effect of Demographic Characteristics and Oral Motor Function on Cognitive Function

To determine whether oral motor function, number of teeth lost, and occlusal force affected the MoCA-K scores after controlling for exogenous variables, a hierarchical regression analysis was performed with educational level and job as control variables (Table 4). Model 1 entered education level and occupation as control variables to determine the effect on MoCA-K; model 2 additionally entered oral–facial function to determine the effect of oral–facial function on MoCA-K after controlling for exogenous variables; and model 3 examined whether occlusal force and number of lost teeth, which have been proven to be related to cognition in previous studies, affected MoCA-K after controlling for general characteristics and oral–facial function. Models 1, 2, and 3 were confirmed to be appropriate regression models and had no multicollinearity problems (tolerance [TOL] ≥ 0.1, variance inflation factor < 10). Model 1 = 0.402, model 2 = 0.575, and model 3 = 0.643, increasing the R-squared change by 0.173 and 0.068, respectively. The significance probability according to the R square F change indicated that the independent variable was statistically significant in explaining the dependent variable after the control variable was entered (*p* < 0.001). The results of the regression coefficient test for model 3 revealed that tooth loss and occlusal force had a statistically significant effect on MoCA-K score(s). The β value for lost teeth was −0.199, indicating that the MoCA-K score decreased as the number of lost teeth increased. The β value for occlusal force was 0.316, indicating that the MoCA-K score increased as the occlusal force increased (F = 11.544, *p* < 0.001) (Table 4).

## 4. Discussion

The present study investigated the relationship between oral motor function (cheeks, lips, tongue, and masseter muscles) and cognition in older adults. The mean (± SD) age of the participants was 73.84 ± 4.5 years, and the proportion of females was 67.3%, which was approximately twice that of males. The mean tongue pressure was 5.78 ± 9.47 kPa, lip pressure was 18.85 ± 4.61 kPa, the dominant cheek pressure was 20.21 ± 4.40 kPa, and the non-dominant cheek pressure was 19.61 ± 4.8 kPa. According to Vanderwegen et al. [28], tongue pressure varies with ethnicity. Consistent with this, the tongue pressures of the participants in this study were lower than those reported by Clark and Salomon [14]. In contrast, in the study by Park et al. [29], which compared differences in the pressure of the lips, cheeks, and tongue according to age and sex in Asian subjects, the lip value in this study was higher, and the pressure of the cheeks and tongue were lower than those of the females and older groups. Unlike previous studies that reported data according to sex and age, this study did not distinguish between them, making it difficult to make an accurate comparison, although it is believed that there were differences in participant height, weight, and body mass index.

In previous studies, general characteristics related to cognition included age, sex, education level, and ethnicity [8,30,31]. Similarly, this study revealed differences in age, education level, and employment status. Cognitive decline is believed to occur with advancing age, and there are differences in cognition depending on education level as well as differences in social activities and environmental stimuli depending on employment status, which may lead to differences in cognition.

In this study, the pressure of the lips and cheeks and the pressure and thickening of the masseter and tongue muscles exhibited a positive correlation with the MoCA-K score; however, the results of the regression analysis revealed no effects. When examining the mechanism by which oral motor function affects cognition, healthy oral motor function can affect cognition in older adults by increasing cerebral blood flow through active mastication [32] or by enabling active social activities through active conversation. According to a previous study, decreased lip movement causes unclear pronunciation and difficulty in conversation [33]. Individuals with high tongue pressure are more socially active than those with low tongue pressure [16], and decreased social activity in older adults can cause cognitive decline [34].

Muscle thickening decreases with age, and decreases in muscle thickening cause a decrease in pressure [3]. The decrease in pressure and thickening of the masseter and tongue and the decrease in the pressure of the lips and cheeks revealed in this study appear to reflect this. However, if this decrease in muscle thickness does not directly cause masticatory difficulties, it may not affect cognition; therefore, it is believed that there was no correlation between oral motor function and cognition in this study. Future studies should aim to identify the oral motor function levels that cause masticatory difficulties and cognitive decline.

In contrast, occlusal force and tooth loss were associated with MoCA-K scores. These results are consistent with previous studies that reported that occlusal force and tooth loss are associated with cognition [24,30,31]. Tooth loss is a common symptom among older adults. Previous studies have reported that tooth loss is associated with oral motor and cognitive functions. When teeth are lost due to a lack of nutrients, such as vitamin B, changes in eating habits can affect cognition [35]. Older individuals with tooth loss exhibit significant shrinkage of gray matter in the brain regions responsible for memory and cognition, such as the hippocampus, caudate nucleus, and temporal lobe [36]. In addition, a decrease in the number of natural teeth can cause significant changes in oral motor structures, such as loss of sensory feedback and decreased muscle tone [37]. This can reduce chewing function, affect dietary preferences, change nutritional status, and, ultimately, affect cognition [38,39]. However, given that some studies have reported that occlusal force is not associated with cognition, further research is needed [40]. The present study aimed to investigate the relationship between oral motor function and cognition, and is more meaningful because it examined the thickening of the tongue and masseter muscles and the pressure on the cheek, for which there is insufficient evidence. 

This study found that oral motor function did not affect cognition. The limitation of this study is the participants were older adults with normal cognitive function. Future research should explore the relationship between oral motor function and cognition in older adults with varying levels of cognitive function, including mild cognitive impairment and dementia. 

## 5. Conclusions

This study examined the relationship between oral motor function and cognition in Korean older adults. Many previous studies have reported that chewing ability and the number of remaining teeth in older adults affect cognition; however, there is insufficient evidence regarding the relationship between oral motor function and cognition. The results of this study showed that the strength of the cheeks and lips, as well as the strength and volume of the tongue and masseter muscles, were significantly correlated with the MoCA-K. However, regression analysis indicated that these correlations were not statistically significant. A limitation of this study is that it only included older adults with normal cognition. Future studies on the relationship between oral motor function and cognition should focus on older adults with varying cognitive levels. Additionally, studies on other variables related to cognition and oral motor, such as social activity and nutritional status, are also necessary.

## Figures and Tables

**Table 1:** Demographic characteristics, cognitive function, and oral motor function of the participants.

Category		Mean ± SD or N (Percentage)
Age (years)		73.84 ± 4.57
Gender	Male	37 (2.7)
	Female	76 (67.3)
Education level	No education	0 (0.0)
	Elementary school	10 (8.8)
	Middle school	38 (33.6)
	High school	56 (49.6)
	University	9 (8.0)
Living with others	Yes	79 (69.9)
	No	34 (38.1)
Smoking	Yes	8 (7.1)
	No	105 (92.9)
Drinking alcohol	Yes	46 (40.7)
	No	67 (59.3)
Work	Yes	17 (15.0)
	No	96 (85.0)
Number of lost teeth		3.04 ± 1.61
Tongue pressure		25.78 ± 9.47
Tongue thickness		28.40 ± 9.51
Occlusal force		469.82 ± 173.41
Masseter thickness at rest		8.16 ± 1.07
Masseter thickness at contraction		9.65 ± 1.45
Cheek pressure (dominant)		20.21 ± 4.40
Cheek pressure (non-dominant)		19.61 ± 4.8
Lip pressure		18.85 ± 4.61
MoCA-K		23.60 ± 4.17

**Table 2:** Cognitive function according to demographic characteristics.

Category		MoCA-K	F or *t*
Gender	Male	23.59 ± 3.86	−0.013
	Female	23.60 ± 4.34	
Educational level a < c, a < d	Elementary school ^a^	20.0 ± 2.94	6.290 *
	Middle school ^b^	22.44 ± 4.71	
	High school ^c^	24.67 ± 3.54	
	University ^d^	25.77 ± 2.99	
Living with others	Yes	25.10 ± 3.44	7.254 *
	No	19.9 ± 3.47	
Smoking	Yes	25.1 ± 2.16	1.879
	No	23.4 ± 4.27	
Drinking alcohol	Yes	23.8 ± 3.79	0.563
	No	23.4 ± 4.43	
Work	Yes	25.29 ± 2.80	2.461 *
	No	23.30 ± 4.31	

^a,b,c,d^ The result of Scheffe post-hoc analysis. * *p* < 0.05.

**Table 3:** Correlation analysis between cognition, age, and oral motor function.

	Age	LT	OF	TP	TT	MT-R	MT-C	CP-D	CP-ND	LP
MoCA-K	−0.557 *	−0.626 *	0.702 *	0.321 *	0.351 *	0.524 *	0.533 *	0.558 *	0.508 *	0.552 *

LT: Lost teeth, OF: Occlusal force, TP: Tongue pressure, TT: Tongue thickness, MT-R: masseter thickness at rest, MT-C: Masseter thickness at contraction, CP-D: Cheek pressure (dominant), CP-ND: Cheek pressure (non-dominant), LP: Lip pressure. * *p* < 0.05.

**Table 4:** Regression analysis of oral cognitive function according to demographic characteristics.

		Model 1			Model 2			Model 3			
	Reference	B	Beta	*p*	B	Beta	*p*	B	Beta	*p*	VIF
LO	Yes	−3.465	−0.380	0.000 *	−2.937	−0.322		−2.004	−0.220	0.011 *	1.912
EL	NE	0.599	0.041	0.747	2.083	0.143	0.240	3.084	0.211	0.065	3.449
Work	Yes	−0.253	−0.022	0.791	−0.338	−0.029	0.709	−0.192	−0.017	0.819	1.396
Age		−0.313	−0.344	0.003 *	−0.223	−0.245	0.023 *	−0.125	−0.137	0.182	2.813
TP					−0.197	−0.449	0.111	−0.132	−0.300	0.253	2.614
TT					0.175	0.400	0.151	0.099	0.227	0.384	2.941
MT-R					0.168	0.043	0.787	−0.404	−0.102	0.498	18.304
MT-C					0.172	0.060	0.696	0.189	0.065	0.645	18.101
CP-D					0.211	0.223	0.181	0.117	0.124	0.424	6.071
CP-ND					0.009	0.010	0.944	0.044	0.051	0.697	5.363
LP					0.179	0.197	0.099	0.152	0.167	0.143	6.425
LT								−0.513	−0.199	0.046 *	4.520
OF								0.008	0.316	0.003 *	3.449
F (*p*)		11.769 (*p* < 0.001)			10.204 (*p* < 0.001)			11.544 (*p* < 0.001)			
*R* ^2^		0.402			0.575			0.643			
adj. *R*^2^		0.368			0.519			0.588			

LO: Living with others, EL: Educational level, NE: No education, TP: Tongue pressure, TT: Tongue thickness, MT-R: Masseter thickness at rest, MT-C: Masseter thickness at contraction, CP-D: Cheek pressure (dominant), CP-ND: Cheek pressure (non-dominant), LP: Lip pressure, LT: lost teeth, OF: Occlusal Force. * *p* < 0.05.

## Data Availability

The data presented in this study are available upon request from the author.
